# Nutrient-Optimized Beef Enhances Blood Levels of Vitamin D and Selenium among Young Women

**DOI:** 10.3390/foods11050631

**Published:** 2022-02-22

**Authors:** Anna Haug, Cees Vermeer, Lene Ruud, Milena Monfort-Pires, Vladana Grabež, Bjørg Egelandsdal

**Affiliations:** 1 Department of Animal and Aquacultural Sciences, Faculty of Biosciences, Norwegian University of Life Sciences, 1432 Aas, Norway; anna.haug@nmbu.no; 2 Cardiovascular Research Institute CARIM (Ret.), Maastricht University, 6229 ER Maastricht, The Netherlands; cees.vermeer@outlook.com; 3 Faculty of Chemistry, Biotechnology and Food Science, Norwegian University of Life Sciences, 1432 Aas, Norway; lene.ruud@nmbu.no (L.R.); milena.monfort-pires@nmbu.no (M.M.-P.); vladana.grabez@nmbu.no (V.G.)

**Keywords:** beef meat, human intervention, young women, vitamin D, selenium

## Abstract

Bovine meat provides healthy nutrients but has also been negatively linked to greenhouse gases and non-communicable diseases. A double-blind intervention study was carried out to compare beef meat from bulls fed with feed supplemented with selenium, vitamin D, E, K (SeDEK-feed), and *n*-3, or REGULAR feed. Thirty-four young healthy women (19–29 years old) consumed 300 g of these beef types per day for 6 days in a cross-over design. Diet registrations, blood samples, anthropometric measurements, and clinical data were collected four times. Both beef diets were higher than their habitual diet in protein, fat, saturated fat, and several micronutrients; contained more vegetables and fewer carbohydrates and were followed by a higher feeling of satiety. The SeDEK beef had higher amounts of selenium, vitamin 25-hydroxyvitamin D3 (25(OH)D3), E, and K (MK4), and increased serum selenium and 25(OH)D3 from the participants’ normal values if they were below 85 µg/L of selenium and 30 nmol of 25(OH)D3/L, respectively. Our study showed that optimized beef increased serum selenium in young women having moderate selenium levels and improved blood 25(OH)D3 in a woman having low to normal 25(OH)D3. Meat should be optimized to increase specific consumer groups’ needs for selenium and vitamin D.

## 1. Introduction

Bovine meat is a valuable food source, providing numerous nutrients and bioactive components that are vital for good health, such as proteins and peptides, B-vitamins, iron, and zinc [1]. Meat is palatable and gives a good satiety feeing and gastronomic pleasure. In the Scandinavian region, utilization of grassland in ruminant husbandry and the production of red meat occurs in areas unsuitable for the production of grain and vegetables. However, red meat has been given considerable negative attention due to concerns for greenhouse gasses and links between red meat and risk of non-communicable chronic diseases including cancer, cardiovascular diseases, diabetes, inflammation, and non-alcoholic fatty liver disease [2,3,4,5,6,7].

In the Western diet, low intakes of vegetables and fiber and a high intake of fat have been reported, while the contents of selenium, iodine, iron, vitamin D, K, and *n*-3 fatty acids are considered to be sub-optimal [8,9,10]. Adequate intake of long-chain *n*-3 fatty acids and a low ratio of *n*-6/*n*-3 fatty acids, for example, have documented positive effects on health parameters [9,11,12]. Micronutrients such as selenium and vitamin E are crucial for defense against oxidative damage and other important biological functions in the body [13,14]. Vitamin K has an established cause-and-effect relationship with the maintenance of bone structure and blood coagulation [15,16].

In Europe, there is a gap between the current and recommended intake of vitamin D [17]. Insufficient vitamin D status is especially prevalent in children/adolescents in northern latitudes due to a lack of adequate sunlight in wintertime [18,19,20]. The vitamin D status in Norway has been evaluated [21]; the report claimed that a significant part of the population has suboptimal vitamin D levels (less than 50 nmol of 25-OH vitamin D/L serum). Vitamin D regulates many vital functions, including immune responses, inflammation, and proliferation associated with total mortality, fractures, falls, and CVD. For elderly persons, having a good vitamin D status is severely important [17,21,22,23].

Throughout the last decade, diets that avoid meat consumption have become more common among youth and young women [24,25,26], as well as vegans [27]. It has been pointed out that the omission of red meat may have negative effects on health, e.g., a lack of iron and vitamins [28,29]. Several of the nutrients in meat, such as selenium, iron, zinc, and some vitamins, have been shown to be more easily digested and absorbed compared to the same micronutrients from plants [30,31,32]. Selenium from meat has been shown to be retained more than twice as compared to plants’ selenium (broccoli) [30]. Nevertheless, attempts to increase human serum concentration using selenium-fortified feed for production animals’ meat have not been successful [33]. Iron as heme has approximately three times the bioactivity of inorganic iron [34]. The dominating form of vitamin D in beef meat is 25(OH)D3 [35], and this form has between 1.5 and 5 times higher bioactivity compared to vitamins D3 and D2 [36,37,38].

Supplementation of the bovine feed with extra nutrients can be an innovative strategy to increase the nutrient content in meat and thereby secure some nutrient and health claims related to compounds of importance to vulnerable groups [39]. Among the nutrients that have been discussed in relation to human health are vitamin D, E, K, *n*-3, and selenium. These five nutrients were deliberately attempted to be increased in the beef by providing a composite feed supplemented with *n*-3, selenium, vitamin D, E, and K to bulls [39], and this feed given to animals increased the concentration of these nutrients in the meat [39]. To our knowledge, there is limited research on how meat from animals that have been fed a diet supplemented with several different nutrients will affect human health markers. When producing bovine meat, all efforts should be made to obtain products with a nutrient composition that may benefit the consumers.

The aim of this double-blind cross-over (6-days × 2) intervention study in young female volunteers was as follows: (1) study the effects of ingesting 300 g of beef a day from bulls fed concentrate supplemented with extra vitamin D, E, K, *n*-3, and selenium (SeDEK-beef) compared to the regular composite feed (REGULAR); (2) compare the ‘300 g beef’ diet with the habitual normal diet of the young healthy participants.

## 2. Materials and Methods

### 2.1. Subjects

A total of 34 free-living young healthy women, varying from 19 to 29 years, with normal weight (BMI 22.9 ± 2.7; mean and standard deviation) participated. The trial was approved by the Norwegian Regional Committees for Medical and Health Research Ethics (REK 2016/620; ES498688; https://helseforskning.etikkom.no/ (accessed on 15 December 2021)) and registered in ISRCTN registry (ID ISRCTN25014465).

### 2.2. Study Design

**Figure 1:**
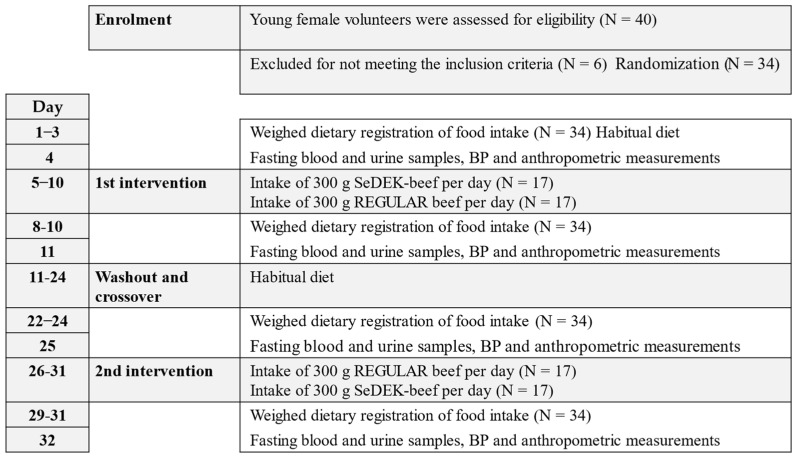
Timeline for the experimental design of the cross-over double-blind study.

The study (32 days) design included a double-blind randomized cross-over intervention study (Figure 1) conducted at the Norwegian University of Life Sciences in Ås, Norway, at a latitude of 60° N during March when skin vitamin D synthesis is minimal.

At baseline, energy content and nutrient composition of the normal, habitual diet were determined by weighted dietary registration (prospective registration) for three days using the “Kostholdsplanleggeren” [40].

The study contained two cross-over intervention periods each for 6 days (Figure 1) with an intake of 300 g raw minced beef meat from the forequarters, in combination with their chosen habitual food items, except fish. The raw minced beef meat corresponds to 240 g cooked beef, as about 20% weight is lost as meat juice during cooking.

The minced beef meat contained about 14% fat, contributing to about 42 g fat per day. The meat was from bulls fed regular control composite feed (REGULAR beef), and from bulls fed composite feed supplemented with vitamins D, E, K, *n*-3, and selenium (SeDEK-beef). Table 1 shows the values for myoglobin, fatty acids, selenium, vitamin D, E, and MK4 for both meats. SeDEK meat also had a lower *n*-6/*n*-3 ratio compared to the REGULAR beef (Table 1). SeDEK beef was a food item containing significant amounts of selenium, vitamin K (mainly as MK4) and vitamin D (mainly as 25(OH)D3) when classified according to the Council of the European Communities [41], as described earlier [39].

The participants received recipes on how to prepare healthy dishes with the meat, preferably adding vegetables. The participants were instructed to look for meat recipes at matprat.no (http://www.matprat.no/sok/#1/all/kj%C3%B8ttdeig/, accessed on 15 December 2021). They were free to use the beef in preparing hamburgers, tacos, or pizzas. The participants were asked to consume the meat in about three meals throughout the day.

The amount of beef given to participants was defined based on power calculation considering that it was a short intervention [42]. Because the amount of meat in the test diet was much higher than their habitual intake, the participants freely changed their diets to adapt to a new pattern with more meat, which included more regular meals and fewer snacks/sandwiches.

During the study, four weighed registrations of the complete food intake were performed, each of them for 3 days (Figure 1); before and during test periods. Then followed a two-week washout period. Samples of blood from the participants were collected in the morning (between 07:00 and 10:00) in fasting state, four times during the study, as well as four registrations of blood pressure, pulse, body weight, and height (only once) of the participants.

Forty young women were recruited to join the study but only 35 could start. The recruitment was conducted at the university by hand-outs and announcements via internet. One person was later removed due to extreme inflammatory markers (see method used below) that aligned with her survey comments on perceived sickness. The remaining 34 participants had an average age of 21.5 years. The 34 participants described themselves as healthy during the study, except a few who said they had a cold/flu for a few days. An a priori power calculation with a two-sided significance level of 5% concluded that 30 participants eating 300 g meat per day were required to observe significant differences in blood levels of selenium, vitamin K, and D. The recruitment was handled by personnel not directly involved in the study or in teaching of students. Exclusion criteria was use of medications, except birth control pills. The participants were asked not to take vitamin-, mineral- or *n*-3 supplements for one month prior to the study and throughout the study period. The participants provided written informed consent before participating. They were informed that they were free to quit the study at any time.

Most participants joined studies in nutrition and food science and had earlier been trained in the method of three-day weighed dietary registration. The participants were informed by a physician if the results from the blood tests were out of the normal reference range.

### 2.3. Anthropometric Measurements

Body weight was measured, without shoes and heavy clothing, to the nearest 0.1 kg using digital scales (Soehnle, Nassau, Germany). Height was measured to the nearest 0.1 cm using a portable stadiometer (Seca 217, Seca, Hamburg, Germany). Body mass index was calculated as weight (kg) divided by the square of height (m).

### 2.4. Blood Analyses

Blood samples were obtained from the antecubital vein for whole blood, serum, and EDTA plasma. Medical personnel trained and authorized for blood sampling performed the sampling at a local medical center. The samples were marked by bar codes and processed within one hour of collection. Serum samples could clot for max 60 min, and plasma samples were put on ice. The tubes were centrifuged at 1500× *g* for 15 min at 4 °C. Whole blood samples, plasma, and serum were stored at −20 °C, and the samples were analyzed at the end of the study, except hemoglobin and blood glucose, which were performed when the blood samples were taken. All blood samples were successfully collected except one blood sample taken from one participant that the phlebotomist failed to draw on one occasion.

#### 2.4.1. Serum Lipids and Other Blood Markers

Total serum triacylglycerol, HDL-cholesterol, LDL-cholesterol, ALT, AST, insulin, C-peptide, and selenium were measured using accredited methods, at a commercial medical laboratory in Norway [43] who also provides the method codes and analytical coefficient of variation.

The inflammatory markers IL-6, IL-8, and IL-1β in EDTA plasma were analyzed using Quantikine ELISA kits Human IL-6, Human IL-8, and Human IL-1β (R&D Systems Europe, Bio-Techne, Abingdon OX14 3NB, UK).

Vitamins K_1_ (phylloquinone) and K_2_ (menaquinones, MK4, MK7, and MK8) in EDTA plasma were analyzed at VitaK laboratories (Maastricht, The Netherlands) by the following method: circulating levels of phylloquinone and menaquinones were measured with a standard HPLC technique using a C18 reversed-phase column and fluorometric detection after post-column electrochemical reduction [44]. Vitamin K1 (G L Synthesis, Worcester, MA, USA) was used as standard [44].

#### 2.4.2. Vitamin D3 and 25(OH)D3 Analyzed in Whole Blood

The analyses were performed at Fødevarestyrelsen, (8520 Lystrup, Denmark) using a 100 µL whole blood where the protein was precipitated using 400 µL 1% formic acid in cold acetonitrile. After mixing and centrifuging (12,000× *g* for 10 min), the supernatant was added to Oasis PRiME HLB 1cc (30 mg, manifold, Extraction Cartridges, Part no. 1860008055, Waters Corporation, Milford, MA 01757, USA) that was conditioned with 1 mL isopropanol/acetonitrile 60/40 plus 1 mL water. The vitamins were eluted with isopropanol/acetonitrile 2% NH3 80/20. Half a ml of the elute was evaporated under weak N2 flow at 25 °C for about 80 min, derivatized by 4-phenyl-1, 2, 4-triazoline-3.5-dione at room temperature for 1–2 h, dissolved in acetonitrile and separated by supercritical fluid chromatography (SFC-MS/MS) on ACQUITY UPC² Xevo TQ-S MS/MS from Waters Milford, MA 01757, USA. The contents of individual components were quantified by the following standards: Vitamin D3 (Cholecalciferol, 97%, ID DLM-8853-D) from Cambridge Isotope Laboratories, Inc. (Tewksbury, MA 01876, USA) and 25(OH)D3 (6,19,19-d3) (ID 705888-1MG) from Sigma-Aldrich A/S (2860 Søborg, Denmark).

#### 2.4.3. 25(OH)D3 in Whole Blood Compared to Serum, a Small Pilot Study

Due to lack of serum, vitamin D3 was analyzed in whole blood. A small pilot study was performed to estimate the conversion factor from blood concentrations to serum 25(OH)D3. Then, blood samples were drawn from five young women, and 25(OH)D3 was analyzed in whole blood and serum. This small pilot study showed a close association between the 25(OH)D3 in whole blood and serum (regression coefficient r = 0.99, *p* < 0.001), and the concentration of 25(OH)D3 was lower in whole blood compared to serum. Data from the five women indicated that 25(OH)D3 in whole blood was 59.2% of the concentration in serum; the mean concentration of 25(OH)D3 in whole blood was 43.7 nmol/L and 73.8 nmol/L in serum. In the current study, the results for 25(OH)D3 were presented as 25(OH)D3 in serum (nmol/L serum), converted from blood values.

#### 2.4.4. Determination of Blood Glucose and Hemoglobin

Determination of glucose and hemoglobin (Hb) was performed directly in blood droplets when the blood samples were taken. Blood glucose was determined by a blood glucose monitoring device, Accu-Chek, Roche, Germany. Hemoglobin was determined by HemoCue Hb 201^+^, (Brønshøj, Denmark) stationed at the medical center. If the results were outside the normal range, the participant was informed on location by the responsible clinical personnel or by the study responsible and advised to contact their personal physician.

### 2.5. Diet Registration

The nutrient intakes were calculated using data from the Norwegian Food Composition database (NFCD) [45] combined with an official diet tool (Kostholdsplanleggeren) [40]. The higher contents in the SeDEK beef of selenium, vitamin D3, and E were adjusted using own data. For the ‘300 g beef diet’, the registration of vitamin D3 intake was calculated as the sum of vitamin D3 plus the biological amount of 25(OH)D3 in the beef; i.e., the concentration of analyzed 25(OH)D3 was multiplied by the factor 5. Information of 25(OH)D3 is not given in the NFCD, and no correction of biological activity was performed for other meat eaten. Information on vitamin K concentration in food is also not given in NFCD and estimates of vitamin K intake were performed by using the Danish Food Composition databases [38] and the corresponding US one [46].

### 2.6. Beef Production

Minced beef meat and fat tissue from quarter, left forepart, from one-year old Norwegian Red bulls reared at the Animal Production Experimental Farm (SHF) at NMBU were used in the study. From 6 months on, six of the bulls were given a regular composite feed, and the other six were given a composite feed supplemented with selenium and vitamin D, E, K, and *n*-3. The intake of feed concentrates, and roughages was registered individually for each animal. The bulls were slaughtered at a commercial slaughterhouse. Details of the study are published [39] and the complete diet is described regarding amounts and sources of ingredients except for the seeds that were from Vestfoldmøllene (Andebuveien 674, 3158 Andebu, Norway). The maximum content of total selenium authorized in feed was used for SeDEK [47].

Meat and fat tissue from each of the 12 animals were removed and minced to a homogenate containing about 14% fat as assessed by QVision (Tomra Systems ASA, Asker, Norway). The minced meat from all the six REGULAR and six SeDEK fed bulls, respectively, generated two batches. The minced meats were vacuum packaged, 300 g in each package, and marked with code 1 or 2 before frozen and stored in the dark at −20 °C for 6 months. The chemical analyses of the meat were performed in the accredited laboratory of the Danish Veterinary and Food Administration (Fødevarestyrelsen, Århus, Denmark). Vitamin K of feed was determined at the National Institute of Nutrition and Seafood Research (NIFES), Bergen, Norway. Muscle myoglobin was assessed as hemin [39].

### 2.7. Nutrient Intake Calculations from Food Composition Databases and Our Own Analyses

The concentrations of selenium, alpha-tocopherol, vitamin K_1_, MK4, vitamin D3, 25(OH)D3, fat, and fatty acids were determined in the forepart beef from REGULAR- and SeDEK-fed animals (Table 1).

The analyzed concentrations of selenium were numerically higher while vitamin D3 and E as alpha tocopherol equivalents (αTE), *n*-3, and -6 fatty acids in the beef were numerically lower than in NFCD [45], respectively. 25(OH)D3 is not reported in NFCD.

### 2.8. Estimation of Satiety in Habitual vs. Diets Holding 300 g Beef per Day

The participants were asked to describe their feeling of satiety (fullness) at four times during the study at days 4, 11, 25, and 32.

### 2.9. Statistical Analyses

Microsoft Office Excel was used for calculations of averages, standard deviations (SD), *t*-tests, and linear regressions. Minitab 18 (https://www.minitab.com/en-us/ (accessed on 15 December 2021) was used for calculations of one-way ANOVA (balanced or GLM) using Tukey’s test as a post-hoc comparison test. Outlier detection was made with Grubbs’ test (used on interleukin data).

Preprocessing of measured variables to fractional changes was also carried out as follows for measured variable *i*: (T_i_ − H_i_)/H_i_ before statistical calculations. A test for values significantly different from zero change was performed using a one-sample Z-test based on the estimated standard deviation obtained for the population mean and standard deviation. A test for the validity of the cross-over design was used [48].

## 3. Results

### 3.1. Baseline Characteristics

The baseline anthropometric data and blood analyses of the 34 participants, standard deviations (SD), min and max values, and reference ranges are shown in Table 2. The baseline characteristics showed that the measured parameters, such as anthropometric registrations and the measured blood analyses at baseline, were within, or close to within, normal ranges (Table 2). One clear exception was their 25(OH)D3 levels.

One participant had hemoglobin (11.4 g/100 mL of blood) below the reference level (11.5 g/100 mL of blood). Four persons had serum C-peptide concentrations (205, 245, 245, and 258 pmol/L serum) below the lower reference level. In the present study, based on the average serum selenium concentrations, participants were close to being selenium deficient [8]. One participant had serum 25-OH vitamin D below 25 nmol/L serum, and 17 of the 34 participants were below 50 nmol/L serum, defined as vitamin D deficiency and the vitamin D lower limit for satisfactory vitamin D status in Norway, respectively [21]. Nine participants had phylloquinone below the reference level of 0.2 µg/L plasma, one participant had MK7 higher than the reference level of 0.8 µg/L, and two participants had MK8 higher than the reference level of 0.45 µg/L plasma.

The baseline diet was as follows: Participant’s habitual diet showed that the intake of products in natura or minimally processed (such as grains, fruits, and vegetables, but also animal products), corresponded to less than 40% of the daily energy intake. Moreover, the intake of fruits and vegetables corresponded to less than 30% of the energy intake from this food group (30% out of 40% of total energy intake), while rice, flour, and pasta represented more than 30%. The intake of non-processed meat was relatively low, representing less than 15% of the energy intake from minimally processed foods (data not shown). The baseline diet contained, on average, about 85 g per day of white-, red-, and processed meat, including liver-pate used as a bread spread, and only 13 g of beef meat. The diet contained in average 37 E% fat (from 27 to 46 E%), 13 E% saturated fat (from 9 to 18 E%), n-6/n-3 ratio was 5.5, 45 E% carbohydrates (from 29 to 62 E%), 18 E% protein (from 12 to 25 E%), 5 E% added sugar (from 1 to 16 E%), 27 g of fiber/day (from 10 to 58 g per day), and 6 g of salt/day (from 4 to 12 g per day).

Their baseline intakes of macro-nutrients were within Nordic recommendations [17] except for the following:

- Iodine; all the 34 persons had a lower intake than the recommended 150 µg per day, the average intake was 69 µg/day; only 46% of the recommended intake. The lower intake level is 70 µg per day [17], and 23 participants were below this level;
- Vitamin D; 29 of the 34. The lower intake level is 2.5 µg per day [17], and 9 of the participants were below this level;
- Iron; 28 of the 34 participants had a lower intake than the recommended 15 mg per day [17], and the average intake was 10.7 mg per day. The lower intake level was 9 mg per day, and 13 of the participants were below this level;
- Selenium: 17 of the 34 participants had a lower intake than the recommended 50 µg per day, and the average intake was 52 µg per day. The lower intake level is 9 mg per day [17], and 13 of the participants were below this level.

No recommendations have been given for vitamin K in the Nordic Nutrition Recommendations [17]. EFSA has set the dietary reference value for vitamin K at 70 µg/day for adults [60], but the U.S. Institute of Medicine has concluded that an adequate intake of vitamin K is 120 and 90 µg/day for men and women, respectively [61].

### 3.2. Nutrient Intakes Were Calculated from Databases and Own Analyses

In Table 3, the nutrient intake was based on data reported in NFCD, while the calculated intakes of fat, *n*-3 and *n*-6, vitamin D, vitamin E, and selenium were corrected according to our own analyses of the SeDEK beef and REGULAR beef (Table 1). The vitamin D in Table 3 shows the sum of vitamin D as given in NFCD plus the amount of 25(OH)D3 as analyzed in the SeDEK beef and REGULAR beef, and then multiplied by 5, as suggested by [62], and as performed in Denmark [38]. Since NFCD does not contain 25(OH)D3, the intake of vitamin D shown for the Habitual diet in Table 3 may be underestimated.

Since vitamin K analyses are not given in NFCD, an estimation of the intake of vitamin K was made by us, based on the Danish and US food composition databases [38,46].

The intake of macro- and micronutrients at the baseline registration did not differ from the intake during the two-week washout period. Based on this, the average nutrient intake in these two registrations is presented in Table 3, left segment, named ‘habitual diet’.

### 3.3. Comparison of the ‘300 g Beef per Day’ Diet to the Habitual Diet

#### 3.3.1. Calculated Nutrient Intake of the Two Diets

The nutrient intake of the 300 g beef diet and the habitual diet is presented in Table 3. The beef diets were significantly different from the habitual diet (Table 3). The intake of beef and meat was significantly higher with the ‘300 g beef’ diet; 312 g of beef and altogether 391 g of meat per day versus about 13 g of beef and altogether 85 g of meat per day when consuming the habitual diet. In addition, the intake of vegetables became significantly higher with the beef diets (*p* = 0.02), while the intake of grains was reduced and 66% of the participants reported that they were eating less bread when consuming the beef diet (*p* = 0.08). Higher satiety was reported when ‘300 g of beef’ was consumed, 77% of the participants were significantly fuller (*p* < 0.001), compared to their habitual diet. However, no significant difference in energy intake between the diets was found (*p* = 0.12). The total content of saturated, monounsaturated, and trans fats, as well as protein intake, increased significantly (*p* < 0.001) during the beef intervention. The intake of polyunsaturated fat (*p* = 0.007), *n*-6 fatty acids (*p* = 0.008), and carbohydrates (*p* = 0.005) was significantly reduced when consuming the ‘300 g beef’ diets compared to their habitual diet. The habitual intake of saturated fat was 12.5 E%, and this is higher than the recommended intake (<10 E%). With beef intervention, the average E% from fat and saturated fat were higher (42.7 E% and 17.6 E%) than recommended (40 E% and <10 E%), respectively [15]. The average intake of protein was higher (21.4 E%) than recommended with beef intervention (10–20 E%) [17]. Accordingly, the E% intake from carbohydrates (35.5 E%) was below the recommendation of 45–60 E% [15]. The intake of fiber was 26.7 g with the habitual diet and 23 g/day with the beef intervention; the latter below the recommended intake (≥25 g/day for women [17]).

The intakes of added sugar were 5.7 E% and 4 E% in habitual and beef intervention, respectively, which were within the recommendations (<10 E%) [15].

The calculated intake of micronutrients with the ‘300 g beef’ diet compared to the habitual diet showed an increased intake of total vitamin K (*p* = 0.008), MK4 (*p* < 0.001), niacin (*p* = 0.003), vitamin B12 (*p* < 0.001), iron (*p* < 0.001), zinc (*p* < 0.001), and selenium (*p* = 0.023). The intake of vitamin A (*p* = 0.014), retinol (*p* = 0.028), thiamin (*p* = 0.022), and iodine (*p* = 0.008) were reduced during the ‘300 g beef‘ intervention compared to the habitual diet. The intake of vitamin D numerically increased (*p* = 0.65) with the beef diet compared to the habitual diet.

All of the participants had a habitual diet that did not meet one or more of the nutrients of the recommended intake [17] of iodine, vitamin D, K, iron, and selenium. Following the beef diets, a significant calculated increase in intake of iron, selenium, and vitamin K was shown (Table 3). The calculated intake of iodine worsened following the beef diet (Table 3); the average intake of iodine was 56 µg during the beef intervention. Eating the beef diet had the following calculated consequences:

Iodine: The iodine intake decreased when consuming the beef diet. Beef meat is low in iodine (~2 µg/100 g); i.e., gives minor contribution to the recommended intake of 150 µg/100 g.

Iron: 20 out of 34 participants were still below the recommended intake of 15 mg of iron per day, in contrast to 28 of the 34 participants at baseline.

Selenium: 5 of 34 participants had a lower calculated intake of selenium than the recommended 50 µg per day, in contrast to 17 of the 34 participants at baseline.

Vitamin K: Two participants were below the estimated intake of 70 µg vitamin K per day during the beef intervention, in contrast to 13 participants at baseline/habitual diet.

#### 3.3.2. Measured Changes in Physical and Blood Variables from Habitual Diets to a Diet including 300 g Beef per Day

Participant characteristics, anthropometric values, and serum analyses from the intervention periods are shown in Table 4, with the habitual diet shown in the left part of the table. The mean of two registrations with their SD following the intake of the beef and habitual diets are given with *p*-values for the comparison between the beef and habitual diets.

Body weight, BMI, blood pressure, and pulse were not affected by beef diets for 6 days, compared to the habitual status.

Blood glucose showed a tendency to increase following the intake of 300 g of beef per day (*p* = 0.078) compared to when consuming the habitual diet (Table 4, left part), but insulin showed no difference.

C-peptide and ALT were not affected by following beef diets compared to the habitual diet, while serum insulin and AST could not be properly interpreted since an effect of the presentation order of the two meats was found (Table 4). This could be interpreted as an additional factor that “accidently” co-varied with the test product, and the reason for this may, among other things, be due to intense exercise among some participants on a specific test diet [63].

Serum lipids (triglycerides, LDL cholesterol, and HDL cholesterol) were not significantly affected by the beef diet compared to the habitual diet (Table 4, left part).

The inflammatory variables in plasma were affected; IL-1β/IL-6 (the cardiac inflammation factor) was reduced, while IL-6 and IL-8 were increased when eating the beef diet compared to the habitual diet.

In addition, vitamin D, K, selenium, and hemoglobin were not significantly affected by daily intake of beef diets compared to the habitual diet (Table 4).

**Figure 2:**
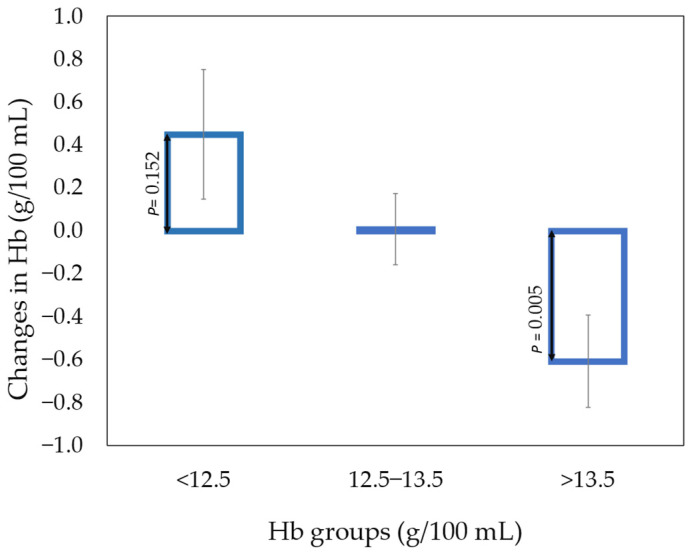
The figure shows the changes in the blood Hb from habitual diet to the intake of 300 g of beef per day. The changes were allocated to three Hb groups Hb according to baseline values (<12.5 mg/100 mL, 12.5–13.5 mg/100 mL, and >13.5 mg/100 mL) where the number in the three groups were 8, 14, and 12, respectively. Mean values with standard errors are given and the p-values were obtained by one-sample Z-analysis indicating probability of obtained values being larger or lower than zero.

Hb showed large variations (ranging from 11.4–15.5 g/100 g of blood at baseline), and the high blood Hb group revealed a significant decrease in Hb blood (Figure 2). The hemoglobin concentration for participants with <12.5 g Hb/100 mL blood (eight of 34 persons) numerically increased by consuming 300 g beef per day compared to eating the habitual diet (Figure 2). The average increase was 0.1 mg Hb/100 mL of blood per day.

Regarding serum selenium, the average concentration at baseline (Table 2) was 84.9 µg/L (1.07 µmol/L), ranging from 65 to 152 µg/L (0.8 to 1.9 µmol/L), but only one person was below 70 µg/L (0.85 µmol/L) and only two persons were above 100 µg/L (1.27 µmol/L) of serum selenium at baseline.

### 3.4. Consuming SeDEK Beef Compared to REGULAR Beef, Calculated and Measured Data

Nutrient intake and study variables following the intake of 300 g of SeDEK beef compared to 300 g of REGULAR beef are shown in Table 3 and Table 4, right parts of the tables, with mean, SD, and *p*-values. Tests based on fractional changes from baseline value, as well as the cross-over test are included in Table 4, right half of the table.

#### 3.4.1. Calculated Nutrient Intake during the Intervention Consuming SeDEK Beef Compared to REGULAR Beef

Consumption of the SeDEK beef compared to REGULAR beef significantly reduced the intake of monounsaturated and n-6 fatty acids in grams (*p* = 0.036 and *p* = 0.032, respectively), although significant differences were not found when presented as E% (*p* = 0.062 and *p* = 0.173, respectively) (Table 3).

An increased intake of vitamin D and MK4 (*p* < 0.001) (Table 3), and selenium (*p* = 0.134) when consuming the SeDEK beef compared to REGULAR beef was found. As shown in Table 1, selenium concentration in the SeDEK and REGULAR beef were rather similar (38 µg and 30 µg/300 g beef, average selenium intake was 67 and 56 µg Se/day, respectively). The beef meat used in this intervention study was relatively rich in Se compared to most retail beef in Norway [35].

The daily intake of vitamin D was 60% higher when consuming SeDEK beef (6.4 µg/day) compared with REGULAR beef (4.0 µg/day), and it was a 75% higher intake of MK4 when consuming SeDEK beef (69 µg/day) compared to REGULAR beef (39 µg/day).

#### 3.4.2. Measured Study Variables following Consuming SeDEK Beef per Day Compared to REGULAR Beef

- Body weight, BMI, Blood Pressure, and Pulse

Table 4 shows anthropometric registrations and study variables for participants consuming 300 g of SeDEK beef compared to 300 g of REGULAR beef per day. When tested for fractional changes from baseline values, significant increases in blood pressure were found (Table 4, right).

- Serum lipids and Other Blood Markers

The concentration of blood lipids was not significantly different following the intake of SeDEK beef compared to REGULAR beef. When tested for fractional change, serum HDL cholesterol significantly increased for the REGULAR beef group (Table 4, right). Since serum LDL cholesterol was affected by the meat presentation order, the interpretation was less clear for LDL cholesterol.

A significant increase in blood glucose using SeDEK beef was observed, independent of the test order of the meats.

When the percentage of change from baseline was calculated (using a one-sample Z-test), the REGULAR beef intake increased hemoglobin concentration.

**Figure 3:**
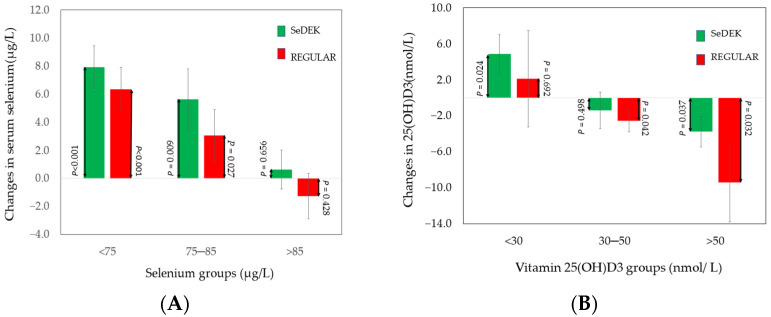
Mean values with standard errors are shown. The *p*-values were obtained by one-sample Z-test indicating probability of obtained values being larger or lower than zero. (**A**) Changes in the absolute serum values for three (<75 µg/ L, 75–85 µg/L, and >85 µg/L) different category groups of initial serum selenium. The average values in the three groups were 72 (N = 8), 80 (N = 13), and 98 (N = 13) µg/L, respectively. (**B**) Changes in the absolute values for three (<30 nmol/L, 30–50 nmol/L, and >50 nmol/L) different category groups of the initial 25(OH)D3 serum values. The average values in the three groups were 23 (N = 3), 40 (N = 15), and 73 (N = 16) nmol/L, respectively.

Serum selenium increased for both beef interventions when the percentage change from baseline was calculated, but the numerical value was highest when SeDEK beef was provided. Participants with serum selenium levels higher than ≥1.15 µmol/L did not have an increase in serum selenium (Figure 3A. The uptake of selenium was larger the lower the initial blood selenium values were. In addition, serum selenium and calculated selenium intake correlated positively (r = 0.503, *p* < 0.01). Serum selenium correlated negatively to heart rate (r = −0.406, *p* < 0.01).

Due to the time of the year (March at 60° N, without sun exposure to increase serum vitamin D), vitamin 25(OH)D3 in the blood declined significantly (*p* = 0.012, GLM model) during the intervention period. The average concentration of 25(OH)D3 in the 34 participants on day 4, 11, 25, and 32 was the following: 55 nmol/L, 51 nmol/L, 49 nmol/L, and 48 nmol/L of serum, respectively. The reference range values for 25(OH)D3 in serum are 50–150 nmol/L [43].

A difference in serum 25(OH)D3 between the two meats was found when the percent/fractional change from baseline was calculated, and the REGULAR beef intake would lead to a significant decline in 25(OH)D3 (Table 4, right and Figure 3B).

The absolute changes expected in 25(OH)D3 serum values depended on initial values (Figure 3B). For serum values above 50 nmol/L, the consumption of both types of meat induced a significant decline in 25(OH)D3. However, for serum values below 50 mmol/L, consumption of SeDEK beef showed no decline, while a significant decrease was detected for REGULAR beef when serum values were 30–50 nmol/L. The decrease of 25(OH)D3 may have occurred due to large variations within the group below 30 nmol/L.

There were no differences in total vitamin K, phylloquinone, MK7, and MK8 in plasma following intake of SeDEK beef compared to REGULAR beef. Plasma MK4, MK5, MK6, MK9, and MK10 were not detected in any of the plasma samples. The percent changes showed a 36–38 percent increase in plasma phylloquinone following both SeDEK and REGULAR meat intake, but the increase was only significant (*p* < 0.05) in the REGULAR beef group.

Vitamin E in beef meat, independent of feed fortification, seems too low to influence calculated intake (Table 3).

- Inflammatory Markers

IL-6 and IL-8 showed small numerical increases with increased intake of meat. When fractional changes were used for analysis, these interleukins increased significantly for SeDEK-beef. Nevertheless, interleukin IL-1β only increased for REGULAR beef per day. We detected a trend (*p* < 0.1) for negative correlations between 25(OH)D3 and IL-6 and IL-8.

## 4. Discussion

### 4.1. The Nature of the Intervention (High Meat and Short Time)

The present cross-over intervention study reported improved vitamin D and selenium status of some groups of young women when consuming beef from bulls supplemented through feeding. High daily intake of beef (300 g of beef in raw weight per day) for 6 days resulted in increased vegetable consumption and did not adversely affect blood lipids, improved Hb levels for some groups, and reduced the cardiac inflammation ratio IL-1ß/IL-6, despite having increased the inflammatory markers IL-6 and IL-8. However, it is unknown whether other environmental factors, such as cold or flu during the winter season or low vitamin D status, could have affected the absolute values of the inflammatory markers. The weeks 3–11 in 2017 had an unusual high prevalence of the winter flu [64].

During the 6 days of 300 g of beef meat consumption, a drastic change in the diet occurred compared to the habitual diet. Apart from the high beef intake, they were free to choose their supplementary food intake and it is noteworthy that they significantly increased the intake of vegetables and reduced the intake of bread. When compared with their habitual diet, the intake of macronutrients during the beef interventions provided more fat and protein and less carbohydrate per day. By joining the study and eating 300 g of beef/day, the volunteers changed their diet towards some of the qualities of a Mediterranean diet, with a higher consumption of vegetables, less bread and refined grains, and moderate consumption of dairy products, which could have influenced some results, such as the lipid profile. The intake of micronutrients was also affected by eating high amounts of meat, more vegetables, and less bread, as shown by the increased intake of vitamin K, B12, niacin, iron, zinc, and selenium. This is in agreement with a study conducted in the US with data from NHANES in which the consumption of bread, as in sandwiches, was associated with fewer vegetable and fruit intakes [65].

Five nutrients (selenium, vitamin D, E, K, and *n*-3 fatty acids) were deliberately attempted to be increased to achieve healthier beef (SeDEK beef), compared to REGULAR beef, but only for selenium and vitamin D were significant blood changes observed. Supplemented feed gave minor differences in content of *n*-6 and *n*-3 fatty acids compared to REGULAR beef, due to bio-hydrogenation of PUFA in the rumen, but no significant effects on blood values were observed. Vitamin E was increased in the SeDEK beef, but the total mean intake of vitamin E was many times higher than the contribution from the beef. The vitamin K3 that was added to the feed was bio-converted to MK4 in the bulls. An intake of 300 g of SeDEK beef could possibly have increased blood levels of MK4, but blood collected after 10 h fasting overnight showed no MK4 and no changes in other MK isomers.

#### 4.1.1. Compliance

None of the 34 volunteers reported difficulties with compliance, although most of them were unfamiliar with such a high daily intake of red meat. The reported data showed high satiety, thus, no difficulties in consumption of the 300 g beef for 6 days × 2.

#### 4.1.2. The Length of the Intervention Study

The 6-day intervention used in this study is shown to be long enough for determination of metabolic responses [42], but may be too short to register stable effects on some of the chosen study variables (such as vitamins, blood lipids, and even hemin/iron).

The present study (6 days) showed that a high intake of beef (three times higher than maximum recommendations) did not negatively affect the blood lipids and improved intakes of iron. On the other hand, beef intake slightly increased some inflammatory markers, and this should be further investigated

### 4.2. Health Outcomes

#### 4.2.1. Serum Lipids

LDL cholesterol is expected to increase with the extra intake of fat, especially saturated fat. However, consumption of 300 g of beef (with 40–45 g of fat, 20 g of saturated fat, and 12 g more saturated fat than the habitual diet) had no significant effect on the increase in the average concentration of serum lipids compared to the habitual diet. This could be due to a counterbalanced effect of increasing vegetable intake, although no increase in fiber intake was detected. Interestingly, only for the REGULAR beef group, the increase from baseline (%) following the diet showed a significant increase in LDL-cholesterol and HDL-cholesterol. However, the crossover p value for LDL cholesterol was 0.021, indicating an effect of the order of the diet. Thus, no strong conclusion can be drawn regarding LDL cholesterol. Although no adverse effects were detected in serum lipid after the beef diets, the short duration of the study (6 days × 2) and the alterations in several nutrients, such as higher intake of protein, selenium, vitamin B’s, D, E, K, and *n*-3, and lower intake of carbohydrates altogether, may have had an effect on the results. Data from a recent meta-analysis indicates that an increased intake of some saturated fatty acids, such as lauric, myristic, or palmitic acid, can increase total, LDL, and HDL cholesterol levels and that the replacement of SFA for PUFA is able to reduce them [66].

#### 4.2.2. Blood Glucose

The blood glucose concentration showed no significant changes from habitual to beef diets, although the intake of SeDEK beef led to a significant percentage (fractional) increase from the baseline. It is a topic of debate if a high protein intake increases blood glucose [67]. It might be suggested that the beef diets were more slowly degraded in the intestine compared to the carbohydrate-rich habitual diet, and a prolongation of the blood glucose levels might be an option.

#### 4.2.3. Micronutrients

- Blood Hb changes with increased meat intake

The increase in iron intake had no effect on average hemoglobin concentration following the intake of ‘300 g beef’ compared to the habitual diet. Only participants having less than 12.5 g of hemoglobin/100 mL and consuming the ‘300 g beef’ diet compared to the habitual diet tended to increase blood Hb after 6 days.

Heme-iron has approximately three times higher bioactivity than inorganic iron [34], and an improvement in heme-status could be expected following the beef diets, since the hemoglobin status of the volunteers was low-to-normal, and an improvement in heme-status has been stated earlier [68]. This result may indicate individual differences between participants. Otherwise, it is possible that a 6-day intervention may not be long enough to obtain significant changes for the low blood Hb group [69]. It has also been shown that hemoglobin synthesis depends on selenium status [70].

- Iodine challenges with increased meat intake

The issue with the low iodine content in meat has recently been approached by feeding supplements of marine algae and seaweeds [71,72].

- Selenium

The increase in serum selenium (%) from baseline levels was significantly higher after the intake of both beef diets, being numerically higher after SeDEK. The increase in serum selenium was high in subjects having serum selenium less than 85 µg/L indicating that beef enriched with selenium (containing more than 10 µg/100 g of beef) may increase serum values in those with lower serum selenium. This agrees with other studies [8].

The selenium concentrations in the two beef types, REGULAR and SeDEK beef (Table 1), were higher than anticipated (5 µg Se/100 g according to the NFCD) [45]. The wheat used for both REGULAR and SeDEK-feed (imported) may have contained higher selenium content than the traditional local wheat. This information was not available during the feed processing, leading to a smaller difference between the REGULAR and SeDEK-feed than intended. A larger difference between the REGULAR and SeDEK feed might have led to larger differences in selenium, possibly giving larger differences in the present crossover intervention study, as has been shown in other selenium supplementation studies [73,74,75]. This shows the importance of feeding domestic animals with a feed with optimal nutritional quality regarding important nutrients such as selenium.

Selenium enriched meat could be a way of securing selenium status for groups having low selenium intake, i.e., individuals that do not consume fish. Ruminant feed enriched with selenium may provide meat products as a natural source of selenium kept at a healthy, beneficial, and harmless level.

- Vitamin D

In general, beef diets contained 5–10 times more 25(OH)D3 than vitamin D3 (Table 1), and the SeDEK beef contained about three times more 25(OH)D3 than REGULAR beef. The importance of 25(OH)D3 in the diet has been assumed to be related to its higher bioactivity compared to vitamin D3.

This double-blind 6-day cross-over intervention study showed for the first time that 25(OH)D3 concentration in the blood improved in young women with a 25(OH)D3 concentration lower than 30 nmol/L when they consumed 300 g of SeDEK beef (containing 0.29 µg of 25(OH)D3 g plus 0.04 ug D3/100 g). When consuming REGULAR beef (containing 0.10 µg/100 g), a significant decrease in blood 25(OH)D3 was found. This suggests that nutrient-optimized beef can enhance vitamin D status.

Intake of SeDEK beef did not improve blood 25(OH)D3 status if serum values were already >50 nmol/l at baseline. An obvious option is to aim towards the higher 25(OH)D3 values published [76]. Our target was set by the highest reported values in the locally produced meat [35,39]. Another possibility is that six days is too short a duration for observing larger differences, or that regulatory processes help to conserve the vitamin D level when the serum concentration is low.

- Vitamin K

Supplemented vitamin K3 added to the bull feed was bio-converted to MK4 in the bulls. Following the intake of 300 g of the SeDEK beef, compared to REGULAR beef, an increase in MK4 concentration in plasma was expected. However, MK4 was not detected at all in plasma in any of the participants. This may be due to its short plasma half-time [77]. Therefore, the effects of intake of MK4 on plasma could not be investigated in this study.

The phylloquinone (vitamin K1) concentration in SeDEK beef and REGULAR beef was nearly identical, and for the participants, the plasma levels of phylloquinone increased to about the same extent (38% and 36% increase) from baseline value following the intake of the two beef types, significantly only for the REGULAR group. The observed increase in plasma phylloquinone may be explained by the increase in consumption of vegetables when the participants were eating the beef diet, as plasma phylloquinone concentration has been shown to reflect the intake of vegetables [54].

With the ‘300 g beef’ diet, total vitamin K intake significantly increased in 88% of participants and reached the recommended intake of 70 µg vitamin K/day.

#### 4.2.4. Inflammation

The present study showed a percent increase from baseline in plasma cytokines with the intake of both beef diets. However, no difference between SeDEK beef and REGULAR beef was shown. The increase in IL-6 and IL-8 presents an important finding that indicates the potential impact of the high amount of beef on the inflammatory response. On the other hand, the reduced IL-1β /IL-6 ratio has been linked to reduced cardiac inflammation and to the expression of COX-2 [78].

## 5. Conclusions

Selenium content above 10 µg/100 g and 25(OH)D3 levels above 0.29 µg/100 g in cattle meat will increase fasting serum values of young females with serum values below 85 µg/L and 30 nmol/L, respectively. Nutrient-rich meat has market advantages for females that were deprived of these nutrients. The study points to the need for a higher awareness among meat producers regarding micronutrients. Even apparently small improvements in meat, as in SeDEK, may be of clinical significance for young women with a challenged nutrient status.

## Figures and Tables

**Table 1:** Measured nutrient contents in minced beef meat; control bulls (REGULAR) and bulls fed nutrient optimized composite feed (SeDEK) [39]. Reproduced with permission from Kaveri Thakuria, Meat Science, Elsevier, 2022.

In 100 g ^a^	REGULAR	SeDEK
	N = 6 Bulls	N = 6 Bulls
Selenium, µg	10.0	12.6
Myoglobin ^b^, g	0.38	0.45
Alpha-tocopherol, mg	0.16	0.65
K_1_, µg	2.1	2.0
MK4, µg	9.1	20.3
Vit D3, µg	<0.01	0.04
25(OH)D3, µg	0.10	0.29
Cholesterol, mg	73.0	57.5
Fat, g	13.6	14.9
C14:0, g	0.36	0.39
C16:0, g	3.35	3.48
C16:1 *n*-7, g	0.37	0.42
C18:0, g	2.59	2.82
C18:1 *n*-9, g	5.01	5.56
C18:1 trans, g	0.43	0.47
C18:2 *n*-6, g	0.27	0.25
C18:3 *n*-3, g	0.059	0.070
C20:4 *n*-6, g	0.032	0.029
C20:5 *n*-3, g	0.006	0.006
C22:5 *n*-3, g	0.017	0.019
*n*-6/*n*-3	3.6	2.9

^a^ Analytical measurement errors are given in [35]. ^b^ hemin-based determination.

**Table 2:** Baseline characteristics of 34 participants; anthropometric values and serum analyses, mean values at baseline, standard deviations (SD), min and max values and reference ranges.

N = 34	Baseline	Min	Max	Ref Values
	Mean ± SD			
Age, years	21.4 ± 2.0	19	29	
Height, cm	170 ± 6	157	180	
Body weight, kg	66.1 ± 8.5	50	88	
BMI, kg/m^2^	22.9 ± 2.7	18	29	
Systolic BP	113 ± 8	94	132	90–130
Diastolic BP	70 ± 7	54	84	60–85
Heart rate	74 ± 13	48	102	60–100
Blood glucose, mmol/L, blood	5.0 ± 0.5	3.9	6.6	3.8–6.1
Insulin, pmol/L serum	72.6 ± 31	24	150	18–173
C-peptide, pmol/L serum	476 ± 146	205	850	270–1290
Selenium µg/L serum	84.9 ± 15.1	65.4	151.8	47–142
AST, U/L serum	18.3 ± 4.5	10	27	<35
ALT, U/L serum	14.7± 5.7	8	35	<45
IL-6, ng/L plasma	3.6 ± 0.8	2.2	6.8	<7 ^a^
IL-8, ng/L plasma	21.5 ± 1.2	19.4	23.6	<16 ^b^
IL-1β, ng/L plasma	12.3 ± 2.6	9.9	19.6	<12 ^c,d^
Hb, g/100 mL blood	13.3 ± 1.0	11.4	15.6	11.5–16
Triglycerides, mmol/L serum	0.93 ± 0.28	0.5	1.6	<2.6
LDL-cholesterol, mmol/L serum	2.7 ± 0.81	0.9	4.8	1.2–4.3
HDL-cholesterol, mmol/L serum	1.6 ± 0.41	1.2	3.0	1.0–2.7
Phylloquinone, µg/L plasma	0.35 ± 0.22	nd	0.8	0.2–0.8 ^e,f,g,h^
MK4, µg/L plasma	n.d	n.d.	n.d.	<0.3 ^i^
MK7, µg/L plasma	0.18 ± 0.14	nd	0.82	<0.8 ^j,k^
MK8, µg/L plasma	0.13± 0.18	nd	0.48	<0.45 ^k^
25(OH)D3, nmol/L serum	54.6 ^l^ ± 19.6	11.8	91.7	50–150

^a^ [49]; ^b^ [50]; ^c,d^ [51,52]; ^e^ [53]; ^f^ [54]; ^g^ [55]; ^h^ [56]; ^i^ [57]; ^j^ [58]; ^k^ [59]; ^l^ Only 6 samples out of 34 reached detection level for vitamin D3, and the vitamin D3 values are not presented.

**Table 3:** The calculated (databases and own values) nutrient intake of 34 participants, after the 3-day food registrations, mean values, and standard deviations (SD). The ‘habitual diet’ represents the mean nutrient intakes at baseline, and the washout period, the ‘300 g beef’ represents the mean nutrient intakes when consuming SeDEK beef and REGULAR beef.

N = 34	Habitual Diet	‘300 g Beef’	*p*	SeDEK-Beef	REGULAR Beef	*p*
	Mean ± SD	Mean ± SD		Mean ± SD	Mean ± SD	
Energy, kcal	1848 ± 336	1971 ± 306	0.117	1894 ± 313	2049 ± 383	0.071
Higher satiety, % of participants	3	77	<0.001	-	-	-
Intake of beef, g	12.7 ± 2.3	312.2 ± 8.4	<0.001	-	-	-
Intake of all meat, g	84.8 ± 17.8	391.0 ± 28.8	<0.001	-	-	-
Intake of vegetables, g	221 ± 145	314 ± 176	0.020	-	-	-
Intake of cereals, g	222 ± 80	186 ± 86	0.080	-	-	-
Fat E%	36.33 ± 6.01	42.73 ± 4.55	<0.001	42.26 ± 5.2	43.53 ± 6.6	0.385
Saturated fat E%	12.53 ± 3.10	17.60 ± 2.69	<0.001	17.65 ± 3.3	17.56 ± 3.7	0.918
Trans unsaturated E%	0.08 ± 0.18	0.45 ± 0.42	<0.001	0.44 ± 0.56	0.46 ± 0.50	0.909
Monounsaturated E%	13.21 ± 3.20	15.71 ± 2.53	<0.001	14.97 ± 3.2	16.44 ± 3.2	0.062
Polyunsaturated fat E%	6.40 ± 1.89	4.56 ± 1.53	0.007	4.24 ± 1.5	4.88 ± 1.7	0.107
Carbohydrate (incl. fiber) E%	45.68 ± 7.11	35.54 ± 5.13	<0.001	36.15 ± 5.8	34.94 ± 6.7	0.429
Added sugar E%	5.65 ± 4.98	3.97 ± 3.34	0.105	4.03 ± 4.4	3.88 ± 3.7	0.881
Protein E%	18.12 ± 3.67	21.37 ± 2.30	<0.001	21.53 ± 3.0	21.21 ± 2.3	0.620
Fat, g	73.7 ± 21.1	91.5 ± 15.8	<0.001	90.0 ± 15.9	95.0 ± 21.1	0.128
Saturated fat, g	25.08 ± 8.41	37.05 ± 6.05	<0.001	36.1 ± 6.5	38.0 ± 9.2	0.333
Trans unsaturated fat, g	0.51 ± 0.33	1.07 ± 0.25	<0.001	1.01 ± 0.3	1.14 ± 0.4	0.095
Monounsaturated fat, g	26.85 ± 8.73	34.62 ± 6.27	<0.001	31.91 ± 6.0	35.92 ± 9.2	0.036
Polyunsaturated fat, g	13.39 ± 5.71	10.07 ± 3.98	0.007	9.07 ± 3.8	11.10 ± 4.8	0.055
*n*-3, g	2.43 ± 1.99	1.76 ± 1.82	0.151	1.63 ± 1.9	1.94 ± 1.9	0.599
*n*-6, g	9.51 ± 3.35	7.46 ± 2.78	0.008	6.65 ± 2.4	8.28 ± 3.5	0.032
*n*-6/*n*-3	5.86 ± 3.02	5.64 ± 1.80	0.722	5.29 ± 1.9	5.99 ± 2.7	0.220
Cholesterol, mg	281 ± 134	262 ± 97	0.514	249 ± 107	276 ± 123	0.341
Carbohydrate (incl. fiber) g	219 ± 47.8	185.3 ± 46.9	0.005	178 ± 51	193 ± 60	0.263
Starch, g	108.5 ± 29.7	95.3 ± 33.4	0.089	91.6 ± 35	99.0 ± 45	0.458
Mono- and disaccharides, g	76.2 ± 35.0	62.0 ± 24.3	0.057	60.8 ± 25	63.3 ± 31	0.714
Sugar, added, g	26.6 ± 25.1	18.1 ± 15.3	0.094	16.63 ± 16	19.49 ± 17	0.498
Fiber, g	26.7 ± 10.8	23.1 ± 9.8	0.149	22.33±10	23.83 ± 11	0.560
Protein, g	81.4 ± 21.0	104.9 ± 14.9	<0.001	102 ± 18	108 ± 16	0.214
Salt, g	6.07 ± 2.11	6.18 ± 2.47	0.843	6.66 ± 3.2	5.69 ± 2.7	0.175
Vitamin A, (RAE)	1227 ± 1327	634 ± 349	0.014	593 ± 436	675 ± 491	0.469
Retinol, µg	648 ± 1007	250 ± 210	0.028	217 ± 205	283 ± 383	0.379
Beta-carotene, µg	4217 ± 3562	3885 ± 3589	0.704	3858 ± 4350	3913 ± 4252	0.957
Vitamin D ^a^, µg	4.91 ± 3.26	5.20 ± 1.78	0.650	6.39 ± 1.7	4.01 ± 2.9	<0.001
Vitamin E, αTE	13.16 ± 4.75	11.49±3.70	0.112	11.38 ± 3.3	11.61 ± 5.0	0.821
Vitamin K (total) ^b^ µg	100.71 ± 83.5	157.7 ± 88.0	0.008	155.6 ± 94	159.9 ± 119	0.869
MK4, µg	10.57 ± 7.05	54.15 ± 5.63	<0.001	69.29 ± 2.8	39.00 ± 10.2	<0.001
Thiamin, mg	1.53 ± 0.46	1.26 ± 0.49	0.022	1.29 ± 0.6	1.23 ± 0.5	0.648
Riboflavin, mg	1.50 ± 0.42	1.50 ± 0.34	0.923	1.45 ± 0.4	1.55 ± 0.4	0.288
Niacin, mg	18.28 ± 6.05	22.15 ± 3.91	0.003	21.38 ± 3.8	22.92 ± 4.7	0.141
Vitamin B6, mg	1.80 ± 0.57	1.72 ± 0.49	0.527	1.64 ± 0.5	1.79 ± 0.6	0.243
Folate, µg	309 ± 127	282 ± 107	0.336	261 ± 91	302 ± 144	0.173
Vitamin B12, µg	5.16 ± 2.22	7.10 ± 1.31	<0.001	6.96 ± 1.5	7.24 ± 1.7	0.480
Vitamin C, mg	104.4 ± 67.1	119.5 ± 70.8	0.370	120.5 ± 95	118.6 ± 72	0.929
Calcium, mg	835 ± 328	737 ± 254	0.169	726 ± 357	748 ± 327	0.793
Iron, mg	10.77 ± 3.20	14.08 ± 2.90	<0.001	13.88 ± 3.0	14.28 ± 3.5	0.616
Sodium, mg	2405 ± 871	2454 ± 1016	0.833	2644 ± 1297	2263 ± 1074	0.192
Potassium, mg	3210 ± 1071	3440 ± 979	0.358	3382 ± 913	3499 ± 1242	0.662
Magnesium, mg	337 ± 115	326 ± 103	0.693	313 ± 99	340 ± 123	0.316
Zinc, mg	9.76 ± 2.16	20.55 ± 2.08	<0.001	20.11 ± 2.5	20.98 ± 2.6	0.162
Selenium, µg	49.12 ± 25.69	61.61 ± 28.54	0.023	66.82 ± 37.9	56.41 ± 12.8	0.134
Copper, mg	1.31 ± 0.53	1.30 ± 0.42	0.955	1.24 ± 0.4	1.36 ± 0.5	0.330
Phosphorous, mg	1509 ± 350	1573 ± 307	0.424	1527 ± 334	1620 ± 389	0.296
Iodine, µg	76.38 ± 34.81	55.72 ± 27.45	0.008	51.33 ± 29	60.10 ± 37	0.283

^a^ The vitamin D concentration in SeDEK and REGULAR beef is the sum of analyzed values for D3 + (25(OH)D3 × 5). In the NFCD [45], only vitamin D is given. ^b^ Vitamin K and MK4 are not given in the NFCD (Matvaretabellen.no) and the intake was estimated using values from ‘the Danish Food Composition Database [38] and from the US Food Composition databases [46]. Total K is the sum of K1 + MK4.

**Table 4:** Participant characteristics, anthropometric values, and serum analyses of 34 participants. Part I shows habitual and the ‘300 g beef’ meat diets. Part II shows the 2 different meat diets in absolute values (upper part of cell) and as changes (lower part of cell). Part III tests for an effect of the 2 meat products’ sequence.

	Part I			Part II			Part III
Variable (N = 34)	Habitual Diet	300 g Beef	*p* ^a^	SeDEK-Beef	REGULAR-Beef	*p*	Cross Over
	Mean ± SD	Mean ± SD		Mean ± SD	Mean ± SD		*p* ^b^
Body weight, kg	66.35 ± 8.58	66.64 ± 8.64	0.895	66.45 ± 8.71	66.47 ± 8.83	0.991	0.372
				0.001 ^c^ ± 0.011	0.002 ± 0.010	0.681	
BMI	22.99 ± 2.75	23.04 ± 2.79	0.944	23.03 ± 2.80	23.04 ± 2.82	0.993	0.839
				0.001 ± 0.010	0.002 ± 0.010	0.666	
Systolic BP	113.3 ± 7.16	115.8 ± 7.17	0.153	116.4 ± 9.02	115.4 ± 7.32	0.617	0.204
				0.035 ** ± 0.081	0.018 ± 0.076	0.372	
Diastolic PB	70.4 ± 5.46	71.3 ± 5.88	0.544	71.3 ± 7.49	71.4 ± 5.95	0.957	0.175
				0.011 ± 0.120	0.025 * ± 0.072	0.543	
Pulse	74.18 ± 9.87	75.29 ± 8.88	0.625	76.50 ± 11.46	74.35 ± 10.26	0.419	0.378
				0.030 ± 0.162	0.025 ± 0.146	0.884	
Blood glucose, mmol/L blood	5.19 ± 0.52	5.39 ± 0.42	0.078	5.47 ± 0.58	5.31 ± 0.41	0.190	0.125
				0.058 ** ± 0.127	0.039 ± 0.122	0.538	
Insulin, pmol/L serum	70.10 ± 25.17	74.02 ± 30.24	0.564	74.75 ± 31.65	75.44 ± 30.44	0.928	0.038
				0.113 ± 0.435	0.274 ± 0.610	0.221	
C-peptide, pmol/L serum	459 ± 111	471 ± 138	0.687	492 ± 156	462 ± 124	0.386	0.413
				0.078 ± 0.290	0.085 ± 0.318	0.921	
AST, U/L serum	19.13 ± 5.23	17.59 ± 4.86	0.212	17.62 ± 5.10	18.03 ± 5.79	0.758	0.007
				0.047 ± 0.212	0.052 ± 0.207	0.914	
ALT, U/L serum	15.41 ± 6.64	15.50 ± 6.41	0.955	15.53 ± 6.45	15.91 ± 8.16	0.833	0.171
				0.023 ± 0.191	0.040 ± 0.215	0.741	
AST/ALT	1.31 ± 0.26	1.22 ± 0.26	0.139	1.22 ± 0.34	1.21 ± 0.25	0.905	0.030
				−0.059 ± 0.195	0.076 ± 0.178	0.702	
IL-1β, ng/L plasma	11.3 ± 2.7	10.5 ± 3.0	0.085	10.7 ± 3.6	10.3 ± 2.2	0.511	0.514
				−0.065 ± 0.245	0.089 *** ± 0.141	0.638	
IL-6, ng/L plasma	3.6 ± 0.8	4.1 ± 1.3	0.038	4.2 ± 1.4	4.0 ± 1.2	0.556	0.455
				0.119 * ± 0.296	0.051 ± 0.220	0.294	
IL-8, ng/L plasma	22.3 ± 2.0	23.2 ± 2.5	0.037	23.4 ± 2.8	23.0 ± 2.2	0.519	0.252
				0.049 *** ± 0.074	0.027 * ± 0.071	0.221	
IL-1β/IL-6	3.3 ± 1.1	2.7 ± 0.7	0.001	2.8 ± 0.8	2.6 ± 0.6	0.500	0.379
				−0.016 ± 0.301	0.014 ± 0.220	0.972	
Hb, g/100 mL blood	13.17 ± 0.90	13.23 ± 0.92	0.755	13.13 ± 1.14	13.32 ± 0.90	0.432	0.350
				−0.016 ± 0.064	0.030 * ± 0.078	0.010	
Triacylglycerol, mmol/L serum	0.89 ± 0.27	0.89 ± 0.29	0.945	0.89 ± 0.26	0.90 ± 0.34	0.850	0.616
				0.033 ± 0.262	0.025 ± 0.220	0.892	
LDL-cholesterol, mmol/L serum	2.62 ± 0.78	2.74 ± 0.86	0.556	2.72 ± 0.85	2.81 ± 0.85	0.638	0.021
				0.043 ± 0.169	0.069 ** ± 0.135	0.492	
HDL-cholesterol, mmol/L serum	1.62 ± 0.37	1.63 ± 0.38	0.950	1.65 ± 0.34	1.67 ± 0.41	0.864	0.227
				0.015 ± 0.098	0.043 ** ± 0.072	0.192	
Selenium, µg/L serum	83.6 ± 12.8	87.0 ± 10.2	0.093	87.9 ± 9.0	86.1 ± 11.4	0.472	0.410
				0.057 *** ± 0.085	0.032 * ± 0.079	0.216	
Phylloquinone, µg/L plasma	0.33 ± 0.22	0.39 ± 0.32	0.410	0.40 ± 0.46	0.40 ± 0.38	0.989	0.302
				0.38 ± 1.15	0.36 * ± 0.97	0.938	
MK7, µg/L plasma	0.19 ± 0.12	0.17 ± 0.11	0.436	0.16 ± 0.11	0.18 ± 0.17	0.589	0.578
				−0.125 ± 0.402	−0.024 ± 0.556	0.456	
25(OH)D3, nmol/L serum	52.0 ± 18.1	49.2 ± 16.8	0.512	50.4 ± 17.2	48.4 ± 16.6	0.630	0.255
				−0.012 ± 0.108	−0.064 ** ± 0.131	0.088 ^d^	

^a^ The *p* represents *t*-tests; ^b^ The *crossover p* reveals probability of an effect of the presentation order [48]. ^c^ The fractional changes from habitual (H_i_) to each 300 g beef test diet intervention (T_i_) are calculated ((T_i_ − H_i_)/H_i_) and the significance (calculated by *t*-test) is indicated as: * *p* < 0.05 ** *p* < 0.01 and *** *p* < 0.001); ^d^ This number was corrected (GLM analysis) for the effect of intervention week.

## Data Availability

The data presented in this study made available https://archive.sigma2.no/pages/public/datasetDetail.jsf?id=10.11582/2022.00007, (accessed on 15 December 2021).
